# When the legs speak for the lungs

**DOI:** 10.1093/sleep/zsaf349

**Published:** 2025-11-01

**Authors:** Mauro Manconi, Raffaele Ferri, Mathias Baumert

**Affiliations:** Sleep Medicine Unit, Neurocenter of Southern Switzerland, EOC, Regional Hospital of Lugano, and Faculty of Biomedical Science, Università della Svizzera Italiana, Lugano, Switzerland; Department of Neurology, Bern University, Bern, Switzerland; Sleep Research Centre, Oasi Research Institute-IRCCS, Troina, Italy; Discipline of Biomedical Engineering, School of Electrical and Mechanical Engineering, Adelaide University, Adelaide, Australia

Over the past 15 years, leg movement activity during sleep has been extensively characterized, with particular attention paid to periodic limb movements (PLMs), revealing several key physiological and clinical aspects [1, 2], including their distribution across sleep stages and throughout the night, the typical inter-movement interval pattern [3], and their marked responsiveness to dopaminergic agents [4, 5]. Moreover, the refinement of scoring criteria allowed clear differentiation between PLM, isolated leg movements (ILMs), short-interval leg movements (SILMs), and respiratory-related leg movements (RRLMs) [6]. What truly caught the attention of clinical research, however, was the tight temporal coupling of motor events with cortical and autonomic arousals [7, 8]. Leg movements may not only accompany sleep disruption but may also contribute to sleep fragmentation and sympathetic activation, potentially increasing cardiovascular risk [9, 10]. Although a causal pathway has yet to be delineated, longitudinal studies suggest elevated PLM may be associated with higher cardiovascular risk, possibly by contributing to chronic hypertension [9]. Conversely, SILM, ILM, and RRLM have historically received much less attention, despite being linked to greater arousability [11, 12].

In this issue of *Sleep*, Esmaeili et al. [13] present data from the MrOS Sleep Study, analyzing 571 older men, to determine whether the frequency of RRLM predicts long-term mortality and cardiovascular event incidence. After adjusting for traditional cardiovascular risk factors and sleep-disordered breathing severity, higher RRLM indices were associated with an increased risk of all-cause mortality over an 11-year follow-up.

Beyond this primary finding, the study confirmed two observations previously reported by other authors: first, individuals with frequent PLMs and obstructive sleep apnea (OSA) also tend to exhibit higher RRLM indices [14]; second, respiratory events coupled with RRLM are longer and associated with deeper oxygen desaturations than those without RRLM [15]. The former supports the hypothesis of a possible genetic or neurophysiological predisposition to motor hyper-responsiveness. In contrast, the latter reinforces the idea that the magnitude of cortical and motor responses increases with respiratory event severity.

Importantly, Esmaeili et al. [13] observed that while RRLM frequency predicted all-cause mortality, it did not predict incident cardiovascular disease after adjustment for apnea–hypopnea index (AHI), suggesting that the physiological mediator of cardiovascular risk remains primarily the respiratory disturbance itself, rather than the leg movements per se. RRLM may therefore act as a *proxy* for apnea severity and the associated sympathetic stress rather than as an independent pathophysiological driver.

In contrast, PLM predicted neither mortality nor cardiovascular outcomes. While this finding aligns with the known differences in autonomic and cortical reactivity, i.e. heart rate and EEG-arousal responses are typically stronger during RRLM [16], SIL,M, or ILM than during PLM [12], it appears inconsistent with earlier analyses of the MrOS cohort, in which elevated PLM index had been linked to cardiovascular disease and hypertension [17, 18]. The discrepancy likely reflects methodological differences, particularly in how RRLM were handled within the PLM scoring window, and associated condition (apnea vs. no apnea), raising the possibility that unrecognized RRLM may have confounded previous associations.

Indeed, because PLM frequency is inversely related to RRLM frequency, accurately separating these two movement types is critical. In addition, this seems to indirectly support the idea that RRLM may reflect, at least in part, PLM captured and synchronized within the rhythm of respiratory events [19, 20]. By applying extended scoring criteria (±5 s window around event termination), the present study underscores how definitional rigor can materially alter epidemiological conclusions. It may even warrant re-evaluating prior studies that used narrower definitions (e.g. ±0.5 s), which likely underestimated the prevalence of RRLM and conflated them with PLM. This conceptual and methodological refinement represents a major contribution of Esmaeili et al.’s work [13]. Despite this, evidence suggests using a time window for classifying leg movement in RRLM to from 2.5 s before to 10.25 s after the end of the respiratory event [21].

The strengths of this study include its long follow-up, rigorous scoring of RRLM, and exclusion of individuals with pre-existing cardiovascular disease, which enhance the reliability of the observed associations. Limitations include the absence of data on restless legs syndrome, which might influence movement propensity, and the lack of information on respiratory effort-related arousals, which could further clarify the total arousal burden and the absolute number of RRLM. Further, the selection criteria, i.e. no prior diagnosis of cardiovascular disease, may be atypical for the population and limit the generalizability of the approach for risk assessment.

In the broader context, these findings advance our understanding of sleep-disordered breathing beyond traditional metrics such as AHI. They highlight RRLM as a potentially integrative marker of both respiratory and autonomic stress within the same AHI range.

From a clinical standpoint, the practical implications depend largely on how causality is ultimately clarified. If the current findings are confirmed, additional interventions targeting RRLM are unnecessary in patients with obstructive sleep apnea who adhere to CPAP or other effective therapies, since the resolution of apneas inherently eliminates RRLM, which, by definition, occur only in temporal association with respiratory events. Quantification of RRLM becomes more clinically relevant for patients who do not tolerate CPAP and continue to experience frequent RRLM. For these individuals, it will be crucial to investigate whether dopaminergic agonists, already known to suppress PLM [4, 5], are also effective in reducing RRLM and potentially mitigating their physiological impact. Conversely, if the causal role of RRLM in mortality is not confirmed, their clinical value would shift toward a marker of disease severity rather than a therapeutic target, helping to phenotypically stratify OSA patients and to emphasize the urgency of adequate treatment initiation or optimization.

Future studies should aim to replicate these results in more diverse populations, including women and younger adults, and determine whether reducing RRLM frequency through therapies that stabilize respiration, lower arousability, or modulate autonomic output translates into better cardiovascular outcomes. From a practical perspective, routine quantification of RRLM in polysomnographic reports could enrich clinical risk stratification at minimal additional cost. However, leg movements are often not recorded by home sleep apnea test devices, limiting the approach’s reach.

In essence, this study reminds us that the briefest leg movements may carry long-lasting messages about the body’s resilience and vulnerability, and that RRLM definition details can reshape our perception of the physiological links between sleep and health.

## Disclosure statement

*Financial disclosure:* None of the authors have financial disclosure to declare.

*Non-financial disclosure:* None of the authors have non-financial disclosure to declare.
